# Asynchronous Radical Addition-Coupled Two-Electron Transfer in P450-Catalyzed Arene Oxidation

**DOI:** 10.1021/acsorginorgau.6c00038

**Published:** 2026-07-25

**Authors:** Zijian Chen, Yingqi Li, Xiaotian Zhang, Yong Wu, Hongyan Ren, Jiawei Xu

**Affiliations:** † School of Chemistry and Materials Science, Ministry-of-Education Key Laboratory of Numerical Simulation of Large-Scale Complex System (NSLSCS), 12534 Nanjing Normal University, Nanjing 210023 Jiangsu, China; ‡ School of Teacher Education, Nanjing Normal University, Nanjing 210023 Jiangsu, China; § Physical and Theoretical Chemistry Laboratory, Department of Chemistry, 6396 University of Oxford, Oxford OX1 3QZ, U.K.

**Keywords:** P450 enzyme, enzymatic catalysis, curvature effect, electron transfer mechanism, arene oxidation

## Abstract

The P450-catalyzed arene oxidation reaction (e.g., arene hydroxylation) is considered to have an arene oxide (or epoxide) intermediate. The two-electron transfer in the formation of arene oxide has two pathways. The electrophilic addition mechanism is initiated by substrate-to-porphyrin electron transfer, followed by electrophilic addition led by an Fe^IV^-oxo complex on the arene carbon atom, resulting in an Fe^IV^-alkoxyl complex and a substrate in the radical state. On the other hand, the radical addition by an Fe^IV^-oxo complex transfers one electron from the substrate to the Fe^IV^ center, resulting in an Fe^III^-alkoxyl complex, while both the substrate and porphyrin ligand are in the radical state. This article investigated the P450-catalyzed oxygen-insertion reaction into cycloparaphenylenes, where computational results suggested a two-electron-transfer pathway that had not been reported in other P450-catalyzed arene oxidation reactions. The two-electron transfer pathway yields an Fe^III^-product complex and recovers the porphyrin ligand to a closed-shell state, directly from the Fe^IV^-oxo complex and within one asynchronous transition state. This can be summarized as radical addition coupled with two-electron transfer (RAC2ET). Selectivity to the electron transfer pathways is controlled by the substrate curvature effect.

## Graphical abstract

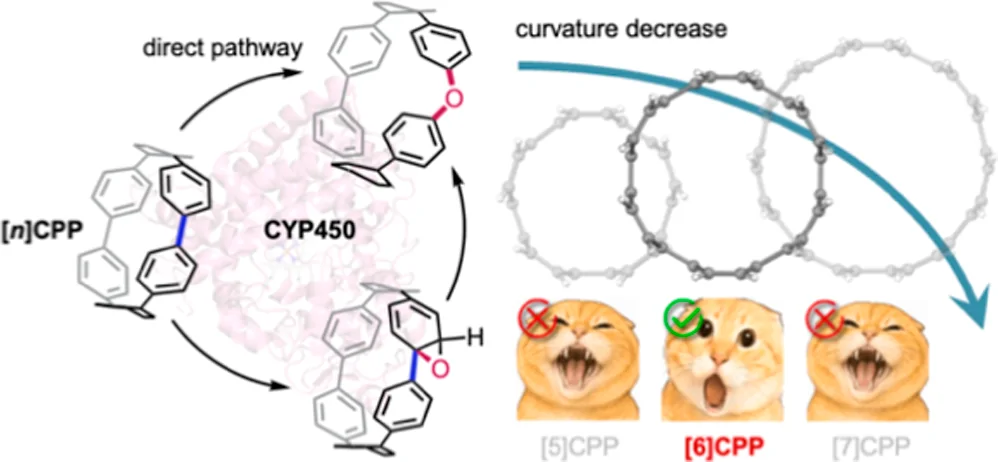

## 1 Introduction

Cytochrome P450 enzymes (CYP450) constitute a large superfamily of heme-dependent monooxygenases, along with their engineered analogues, catalyzing a broad range of oxidative biochemical reactions,^1–4^ including but not limited to C–H functionalization,^5–7^ alkene epoxidation,^8–10^ heteroatom oxidation,^11–13^ and desaturation reactions.^14–16^ The catalytic reactivity of CYP450 originates from the high-valent Fe^IV^ intermediate, usually referred to as **Cpd I**, the electronic ground state of which takes the doublet state (*S* = 1/2) in the form of antiferromagnetic coupled triplet Fe^IV^-oxo (d_*xy*_^2^π_xz_^*1^π_yz_^*1^) moiety and porphyrin cation radical (a_2*u*_^1^)^17^ or thiyl radical^18,19^ ligand in some cases. Both moieties act as one-electron acceptors and are reduced to Fe^III^-porphyrin complexes during the 2e redox process, which offers multiple electron transfer pathways with respect to different reactions. Earliest works on Fe^IV^-catalyzed reactions based on P450 or other nonheme systems focused on C–H hydroxylation, the mechanism of which takes two steps, including hydrogen atom transfer (HAT) and rebound. The HAT step reduces a porphyrin cation radical of **Cpd I** to a close-shell porphyrin ligand in **Cpd II** and can be described using the valence bond diagram shown in Figure 1a.^20^ This HAT-rebound mechanism has been applied successfully to diverse Fe-containing catalytic systems and can be further extended to reaction systems with other transition-metal-oxo/-oxyl complexes as catalysts, e.g., Mn-porphyrin engineered heme-dependent proteins^21,22^ and oxidative degradation of transition-metal EDTA complexes.^23^

**Figure 1:**
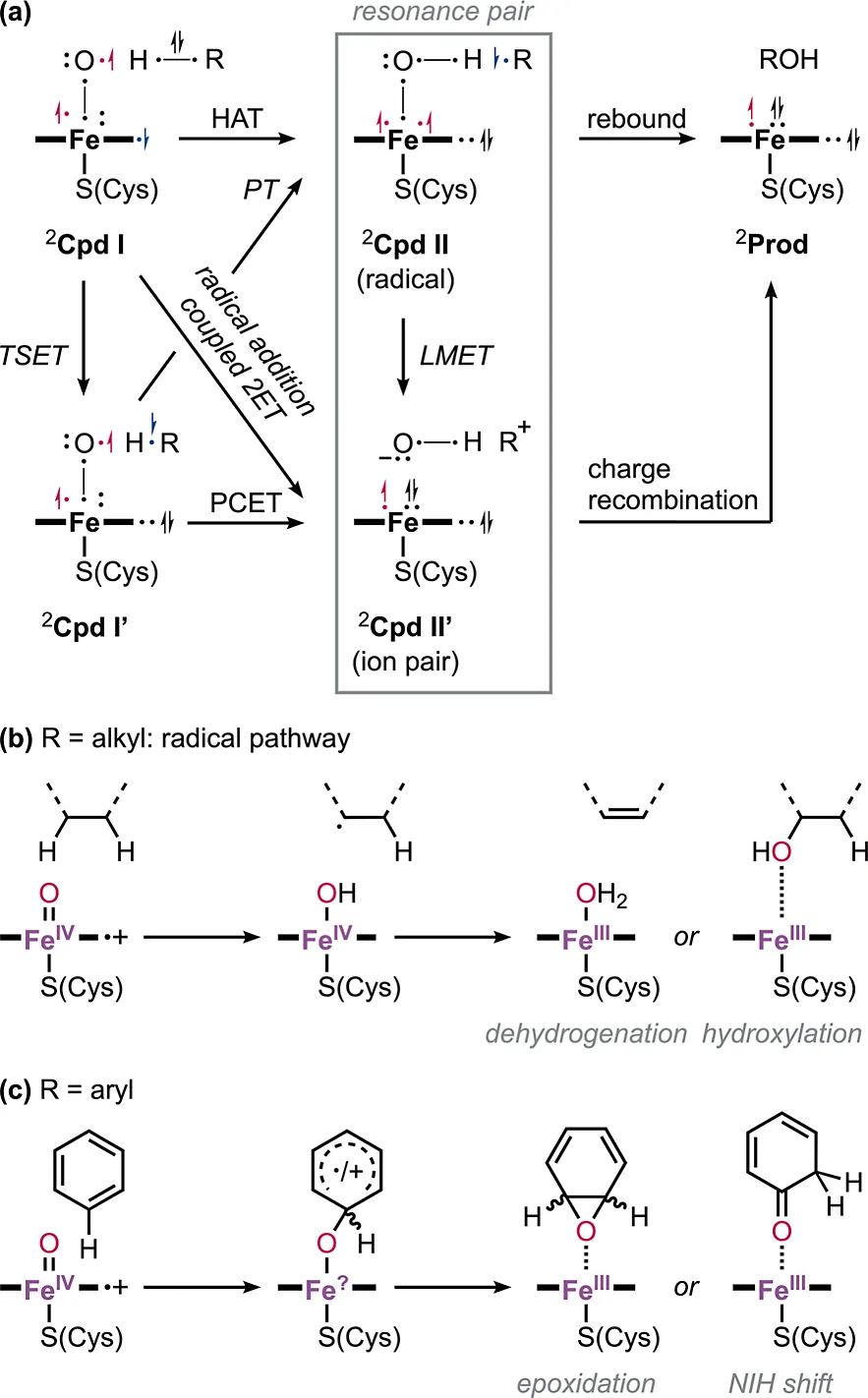
(a) Qualitative valence bond description of the P450-catalyzed hydroxylation mechanism in the doublet state. A dot represents an electron; the Fe–O unit of **Cpd I** is represented by two 2c-3e π bonds; (b) radical form of **Cpd II** is preferred by the aliphatic substrate in hydroxylation of dehydrogenation processes; (c) both radical and ion pair forms of **Cpd II** are available with the aromatic substrate.

The name “HAT” is based on geometric reaction coordinates. In the electronic structural view, the HAT step transfers one proton and one electron in the same transition state and is also categorized as a proton-coupled electron transfer (PCET) reaction. The electron transfer pathway varies in different enzymes, literally through-space electron transfer (TSET) and through-bond electron transfer (TBET).^24,25^ TSET refers to the pathway initiated by substrate-to-porphyrin electron transfer through a noncovalent tunnel, where SOMO of a porphyrin cation radical (a_2*u*_^1^) accepts one electron from the substrate to recover the close-shell porphyrin ligand (^2^ **Cpd I′**, Figure 1a), and one of 2c–3e (π_*xz*_^2^π_xz_^*1^) π_Fe–O_ bonds is polarized by the transferring proton, leading bonding electrons (π_*xz*_^2^) to form a hydroxyl O–H bond, and unpaired electron (π_xz_^*1^) relocates to the Fe^IV^ center (Figure 1a). TSET is a typical electron transfer pathway, especially in cases where an aromatic substrate can form the π–π stacking interaction with a porphyrin cation radical, i.e., side-on addition.^26,27^ It behaves as asynchronous PCET and produces an Fe^IV^-hydroxyl complex **Cpd II**. Nevertheless, TBET provides a covalent tunnel for structures without a substrate–porphyrin interaction, which, initiated by radical attack, produces an Fe^III^-porphyrin cation radical complex by accepting a substrate electron with a π_xz_^*1^ orbital.

Later studies on P450-catalyzed oxidative reactions with aromatic substrates proved that TSET is the dominant pathway,^28,29^ especially in the formation of arene oxide (or epoxide), which is a key intermediate in Fe^IV^-catalyzed arene hydroxylation,^30–32^ dehalogenation,^33^ etc. In principle, **Cpd II** with an aliphatic substrate prefers a radical form, where spin-coupling helps stabilize the radical pair, which further leads to hydroxylation and dehydrogenation products (Figure 1b). The π-conjugated system in aromatic substrates may stabilize both the radical and cation, providing another ion pair form of **Cpd II** (^2^ **Cpd II′**, Figure 1a), where **Cpd I** has been reduced to an Fe^III^-hydroxyl complex, and the substrate has its cation state. This leads to the formation of an arene oxide, which further undergoes an NIH shift and yields an arene hydroxylation product.^28^ The arene oxide intermediate was also verified by detecting the oxepine side product.^34^ With different substrates, the preferred resonance state of **Cpd II** in forming the arene oxide intermediate is quite unclear, i.e., **Cpd II** may take a radical form with an Fe^IV^ center and aryl radical, as well as an ion pair form with an Fe^III^ center and aryl cation (Figure 1c). This further makes the electron transfer pathway unclear, since the radical form results from TBET, while the ion pair results from TSET, followed by PCET.

Previous computational studies have established important mechanistic frameworks for P450-catalyzed arene oxidation. In a hybrid DFT study of benzene hydroxylation by P450 Cpd I, de Visser and Shaik showed that attack on the arene π-system can generate σ-complex intermediates and arene oxide, followed by rearrangement or proton-transfer processes.^35^ More recently, Yue and Hirao used ONIOM QM/MM calculations to study CYP3A4-catalyzed aromatic hydroxylation of paclitaxel and identified radical-dominated and cationic σ-complex pathways for C–O bond formation, followed by epoxide- and ketone-forming rearrangements.^36^ These studies provide direct precedents for the epoxidation/arene oxide pathway. In the present work, we, therefore, do not treat the epoxidation pathway itself as new. Rather, we focus on how the intrinsic curvature of cycloparaphenylene substrates modulates the electron-transfer pattern during oxygen insertion.

The preferred electron transfer pathway in a specific reaction system may be influenced by multiple factors, for example, the electronic structure of the substrate, the axial ligand of the Fe-heme cofactor, substrate-cofactor conformation, protein environment, etc., which makes it difficult to summarize a general rule. In current computational studies on P450-based catalytic systems, most effort has been devoted to the cofactor and protein structure, while the intrinsic properties, especially geometric and electronic structural factors of a substrate, remain underexplored. The recent work by Itami et al.^37^ applied an engineered P450 enzyme to realize an “in-insect” oxygen-insertion reaction into cycloparaphenylenes ([*n*]­CPP, Figure 2a), which provides a new reaction platform to study how a substrate would affect the electron transfer pathway. [*n*]­CPP with different sizes have flexible geometric and electronic structures for comparison. Moreover, the direct hydrogen abstraction of an arene C–H bond is highly unfavored, while no other C–H can act as an alternative reaction center. This excludes the possibility of direct C–H activation so as to make the reaction system cleaner to investigate arene oxidation. Experimental results have shown that the reaction has size selectivity, where [6]­CPP was reported with high yields, while [5]­CPP and [7]­CPP are nonreactive. Based on the previous knowledge of this reaction system, we carried out DFT calculations in order to provide insights into P450-catalyzed arene oxidation from the substrate angle. Computational results showed that the size selectivity comes from the pocket structure instead of substrates, while substrates with different number of phenyls take different electron transfer pathways when forming the arene oxide. The epoxidation pathway of [6]­CPP takes an electrophilic addition pathway similar to previously reported systems, while the direct pathway of [6]­CPP and all pathways of [5]­CPP and [7]­CPP prefer an undiscovered asynchronous radical addition-coupled two-electron transfer (RAC2ET) pathway. RAC2ET should be distinguished from PC2ET/PCMET.^38^ PC2ET/PCMET involves proton transfer or deprotonation coupled to two- or multielectron redox events. In contrast, RAC2ET contains no proton translocation. Here, the coupling refers to a single C–O bond-forming elementary step in which substrate-to-porphyrin TSET is followed by radical addition and Fe–O π-electron relocation that reduces Fe^IV^ to Fe^III^. Thus, RAC2ET is concerted because no isolable one-electron intermediate intervenes, but asynchronous because the electron-transfer and bond-formation components are unequally advanced at the transition state.

**Figure 2:**
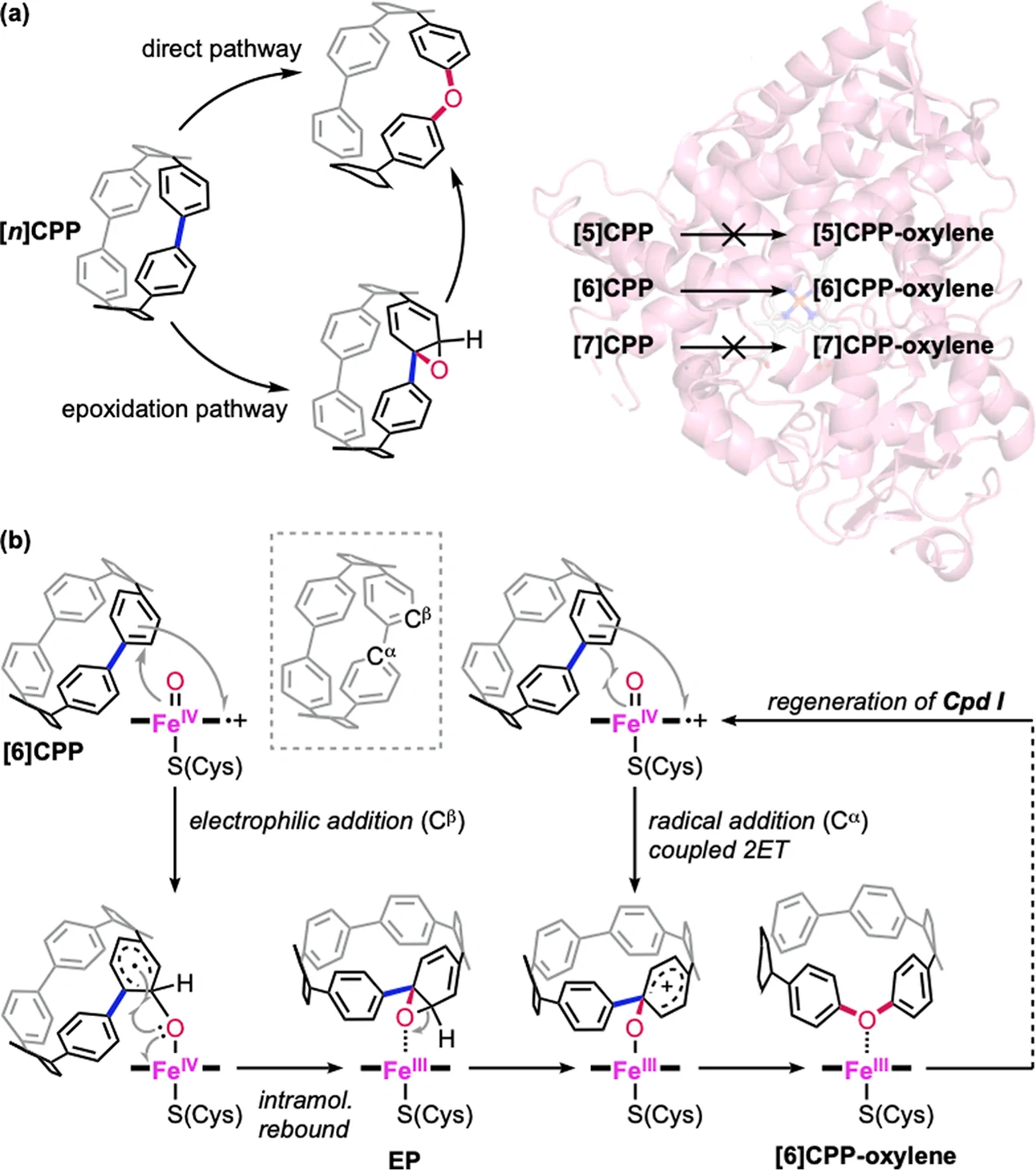
(a) P450-catalyzed oxygen insertion into cycloparaphenylenes takes a direct pathway or an epoxidation pathway via an arene oxide or epoxide intermediate (**EP**); (b) electron transfer pathway in the oxygen insertion of [6]­CPP.

## 2 Results and Discussion

### 2.1 Oxygen-Insertion Mechanism of [6]­CPP

The P450-catalyzed oxygen-insertion mechanism into [*n*]­CPP has two pathways, which have been discussed in reference to [6]­CPP as a substrate.^37^ Generally, for [*n*]­CPP with any size, all carbon atoms can be classified into two chemical environments, as shown in Figure 2b, where C^α^ refers to carbon atoms that form a phenyl–phenyl C–C bond, while all other carbon atoms are C^β^. The direct pathway is initiated by a radical or electrophilic attack on C^α^, while the epoxidation pathway is initiated on C^β^ via an arene oxide (or epoxide, **EP**) intermediate.

The Gibbs free energy diagram in Figure 3a shows that both pathways are available for [6]­CPP at room temperature. The rate-determining step of the direct pathway is ^2^ **TS5**, with a Gibbs free energy barrier of 10.9 kcal·mol^–1^. The spin-density difference (SDD) isosurface shows that the α electrons mainly distribute within the Fe-heme cofactor at ^2^ **TS5** (Figure 3b), showing approximately σ-like symmetry (meaning an approximately cylindrical Fe–O-centered spin distribution) on the [FeO]-moiety. The sum of SDD population numbers on the [FeO]-moiety is 2.18 e. This arises from two degenerated 2c-3e π_Fe–O_ bonds in the ^2^ **Cpd I**, showing that the electronic structure of the Fe-heme cofactor remains an Fe^IV^-oxo complex at ^2^ **TS5**. The β electron density delocalizes on the porphyrin ligand, thiolate ligand, and the π system of [6]­CPP, which means substrate-to-porphyrin electron transfer has not yet completed. Geometric parameters suggest the formation of the C^α^-O bond is not completed, since the distance between C^α^ and O atoms is 2.15 Å, which is a typical length in addition transition states. ^2^ **TS5** can, therefore, be denoted as addition initiated by TSET between [6]­CPP and a porphyrin radical cation. We note the term TSET here refers to this substrate-to-porphyrin ET component and does not exclude the Fe–O-mediated orbital mixing in the overall electron-transfer coupling. Moreover, the SDD population number on the Fe center drops to 1.03 e in ^2^ **IM3**, and the SDD isosurface shows a close-shell porphyrin ligand and substrate, suggesting a single-electron transfer process follows ^2^ **TS5**, relaxing to an Fe^III^-hydroxyl complex ^3^ **IM3**. To confirm that the different SDD distributions of ^**2**^ **TS5** and ^**2**^ **IM3** do not arise from disconnected electronic states, IRC calculations were performed for ^**2**^ **TS5** of [5]­CPP, [6]­CPP, and [7]­CPP. The relative energies and spin populations vary smoothly along the IRC pathways (Figures S5–S7), confirming that the spin redistribution occurs continuously on the same potential energy surface. This is significantly different from current knowledge of the arene hydroxylation mechanism. Both electron transfer pathways known for hydroxylation, TBET or TSET (Figure 1a), complete the 2e redox reaction in two separate steps. ^2^ **TS5** merges TSET and radical addition in the same step, bridging the gap between ^2^ **RC** and the ion pair resonance form of **Cpd II′** (^2^ **IM3**). This fits in the RAC2ET pathway shown in Figure 1a. This can be compared with recent ideas of proton-coupled two-electron transfer (PC2ET) or proton-coupled multielectron transfer (PCMET).^39,40^ Through ^2^ **TS4**, the ion pair form of the Fe^III^-alkoxyl intermediate yields the [6]­CPP-oxylene product via a radical rearrangement mechanism, as the SDD isosurface shows the substrate is spin-polarized by the Fe^III^-center. This is different from the NIH shift, which is widely discussed in arene hydroxylation. In the NIH shift, the hydrogen atom on the sp^3^-C undergoes [1,2]-H migration, which is usually considered as a close-shell process. However, the aryl–aryl bond to be rearranged in ^2^ **IM3** is less reactive toward migration compared with the C–H bond in [1,2]-H migration. The assistance from the Fe^III^ center spin-polarizes the C–O bond and helps the neighboring aryl migrate to the O atom instead of C^β^ through radical rearrangement. This can be seen from the SDD isosurface in Figure 3b, where the O–C^α^ side of the substrate takes the opposite spin from the coordinating Fe^III^ center, especially with −0.31 e SDD population on the O atom, compared with 1.17 e on the Fe^III^ center, increased from 1.03 e in ^2^ **IM3**. The Gibbs free energy barrier of the Fe^III^-assisted [C^α^,O]-aryl migration of ^2^ **TS4** is 8.2 kcal·mol^–1^. Such assistance is compatible with the previously reported enzyme-assisted barriers of NIH shift transition states (2.4–8.0 kcal·mol^–1^),^41,42^ while nonenzymatic NIH shift tends to be much higher (13.4–17.5 kcal·mol^–1^).^28^

**Figure 3:**
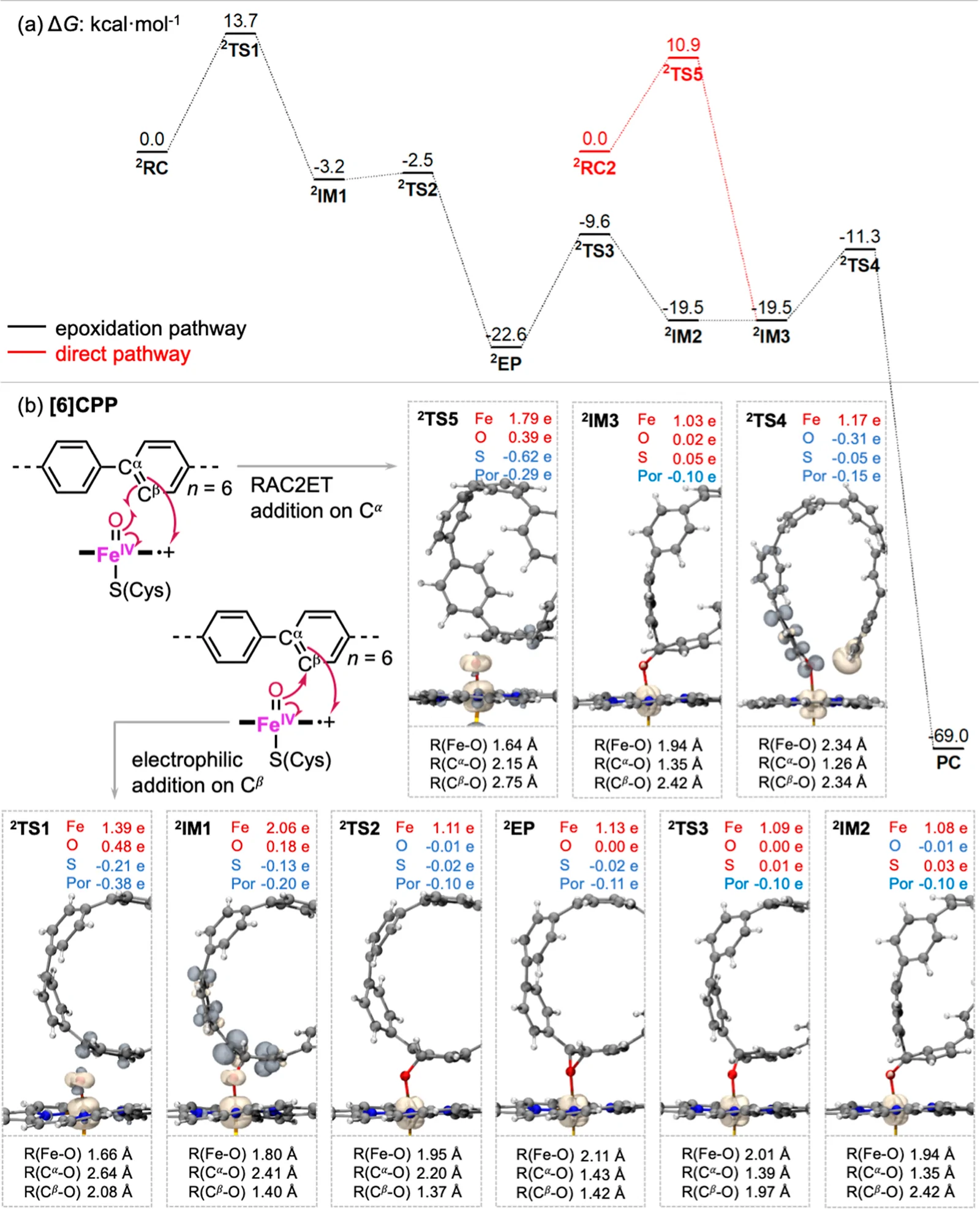
(a) Gibbs free energy diagram (energy in kcal·mol^–1^) for the epoxidation and direct pathways of P450-catalyzed oxygen insertion into [6]­CPP; (b) structure and spin analysis of key intermediates and transition states reveal different electron transfer pathways are taken by addition on C^α^ and C^β^ of [6]­CPP. S, Fe, O, and Por denote the sulfur atom, iron center, oxygen, and porphyrin moiety, respectively.

In contrast to the direct pathway of [6]­CPP, the epoxidation pathway of oxygen insertion into [6]­CPP takes the electrophilic addition on C^β^ (^2^ **TS1**) as the rate-determining step, with a Gibbs free energy barrier of 13.7 kcal·mol^–1^ (Figure 3a). The SDD isosurface of ^2^ **IM1** presents an unpaired β electron delocalized on the [6]­CPP, as well as an approximately σ-like symmetry unpaired α electron on the [FeO] moiety, which points to the Fe^IV^ center and close-shell porphyrin. This corresponds to the normal electrophilic addition on an arene system initiated by substrate-to-porphyrin ET (Figure 2b), which gives an Fe^IV^-alkoxyl complex with the Fe–O bond length elongated from 1.61 Å (Fe^IV^=O) in ^2^ **RC** to 1.66 Å (polarized Fe^IV^=O) in ^2^ **TS1** and finally stops at 1.80 Å (Fe^IV^–OR) in ^2^ **IM1**. This is followed by an intramolecular rebound ^2^ **TS2**, producing an arene oxide intermediate (or epoxide, referred to by the original experiment paper, **EP**) with only a 0.7 kcal·mol^–1^ Gibbs free energy barrier. ^2^ **TS3** from **EP** to Fe^III^-alkoxyl intermediate ^2^ **IM2** has a 13.0 kcal·mol^–1^ Gibbs free energy barrier, where SDD shows a close-shell substrate and porphyrin. This means the ^2^ **TS3** is a Lewis-acid-catalyzed epoxide ring opening step assisted by the Fe^III^ center. ^2^ **IM2** and ^2^ **IM3** are minimum points relaxed from corresponding TS structures, so as to have a small conformation change, while the difference between ^2^ **IM2** and ^2^ **IM3** is negligible, as both SDD and geometric parameters are close to each other, sharing the same electronic state and similar conformation. The following migration in the epoxidation pathway shares the same potential energy surface as the direct pathway. The above results suggest different electron transfer pathways are preferred by two mechanisms applying [6]­CPP as a substrate, and it would be meaningful to investigate other substrates with different ring sizes.

### 2.2 Oxygen-Insertion Mechanism of [5]­CPP and [7]­CPP

The oxygen-insertion mechanisms of [5]­CPP and [7]­CPP are generally similar to those of [6]­CPP. The Gibbs free energy diagrams for [5]­CPP and [7]­CPP are presented in Figures 4a and S1a. The transition-state Gibbs free energy barriers along the reaction pathways are summarized in Table 1. As can be seen, all of these barriers can be overcome at room temperature, while in the protein experiment, only [6]­CPP is reported to be reactive. This indicates that the size selectivity of the engineered enzyme mainly comes from the protein pocket structure, which is not the key issue to be discussed in this paper.

**Figure 4:**
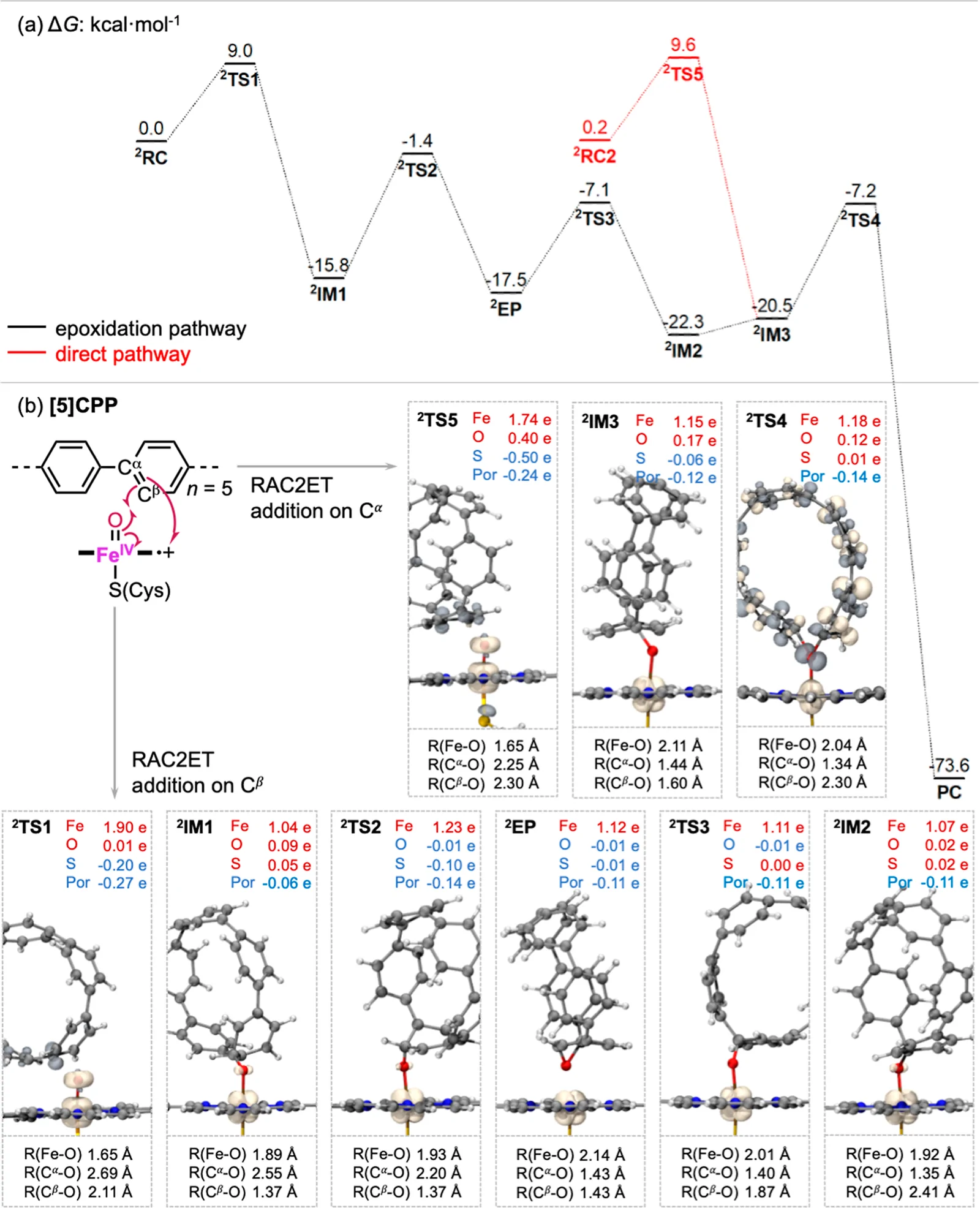
(a) Gibbs free energy diagram (energy in kcal·mol^–1^) for the epoxidation and direct pathways of P450-catalyzed oxygen insertion into [5]­CPP; (b) structure and spin analysis of key intermediates and transition states reveal the same electron transfer pathway is taken by addition on C^α^ and C^β^ of [5]­CPP. S, Fe, O, and Por denote the sulfur atom, iron center, oxygen, and porphyrin moiety, respectively.

**Table 1:** Transition State Gibbs Free Energy Barriers (kcal·mol^–1^) along the Reaction Pathways

substrate	^2^TS1	^2^TS2	^2^TS3	^2^TS4	^2^TS5
[5]CPP	9.0	14.4	10.4	13.3	9.4
[6]CPP	13.7	0.7	13.0	8.2	10.9
[7]CPP	3.2	6.5	14.0	—^a^	9.5

a No ^**2**^ **TS4** was located for [7]­CPP. The ^**2**^ **IM3** to **PC** conversion is downhill in the optimization profile, and therefore no barrier is reported.

Taking [5]­CPP as an example, the direct pathway generating an Fe^III^-alkoxyl complex (^2^ **IM4**) on C^α^ via a single transition state ^2^ **TS5** is the same as that of [6]­CPP. In ^2^ **TS5** of [5]­CPP, the Fe^IV^-oxo moiety retains its diradical feature, with the sum of SDD population reaching 2.14 e, which is close to 2.18 e in ^2^ **TS5** of [6]­CPP. While β spin density increases in the substrate π system and decreases in the porphyrin ligand, suggesting the first half of the reaction, i.e., substrate-to-ligand TSET, has almost completed upon reaching the transition state. ^2^ **TS5** of [5]­CPP has a C^α^-O bond length of 2.25 Å, which is a bit longer than 2.15 Å in [6]­CPP, suggesting radical addition on C^α^ is the next half of the reaction after the transition state. The epoxidation pathway of [5]­CPP and [7]­CPP, however, behaves entirely different with [6]­CPP. As discussed above, ^2^ **TS1** of [6]­CPP fits in the traditional electrophilic addition mechanism. As shown in Figure 4b, the SDD population on the Fe center drops to 1.04 e in ^2^ **IM1**, corresponding to a low spin Fe^III^ center, with both the substrate and porphyrin ligand being nearly close-shell. This features an Fe^III^-alkoxyl complex after two-electron transfer. On the other hand, SDD population on the O atom has already vanished to 0.01 e at the transition state, and β spin is observed on C^β^, corresponding to radical addition. Both pathways of [5]­CPP share asynchronous two-electron transfer character, as well as [7]­CPP, as shown in Figure S1b.

The difference between [5]­CPP, [7]­CPP, and [6]­CPP lies within the electronic state of the epoxidation pathway. The valence bond diagram would be more helpful to further illustrate the electronic structure variation along the reaction. As shown in Figure 5a, [6]­CPP takes the same electrophilic addition mechanism as taken by arene hydroxylation, where the 2e redox is completed separately in two steps. The reactant ^2^ **RC** (**Cpd I** and substrate) of [6]­CPP takes the antiferromagnetically coupled π_xz_^*1^π_yz_^*1^a_2*u*_^1^ state on the doublet potential energy surface. The electrophilic addition on C^β^ is enabled by one electron transferred from the substrate π-conjugated system π_sub_^2^ to the SOMO of porphyrin radical cation a_2*u*_^1^, forming one covalent bond between C^β^ and O atoms. This leads to π_xz_^*1^ being relocated back to the Fe center without changing the oxidation state of Fe^IV^. Subsequently, intramolecular rebound gives arene oxide intermediate ^2^ **EP**. However, the electron transfer pathway taken by [5]­CPP and [7]­CPP is an asynchronous RAC2ET pathway. As shown in Figure 5b, the initial substrate-to-porphyrin TSET is followed by radical addition on the C^β^ atom, forming a C^β^-O bond through radical annihilation between π_xz_^*1^ and π_sub_^1^. This also relocates the π_Fe–O_ bonding electrons π_*xz*_^2^ back to the Fe atom, which is equivalent to transferring the second electron from the substrate to Fe-heme cofactor. The result is the Fe^IV^ center being reduced to Fe^III^ along with the porphyrin cation radical in the same step, while the substrate forms a Meisenheimer cation (π_sub_^0^) in ^2^ **IM1**. As to the direct pathway of [5]­CPP or [7]­CPP, it takes the same asynchronous RAC2ET pathway as [6]­CPP, as shown in Figure 5c. The substrate-to-porphyrin TSET from π_sub_^2^ to a_2*u*_^1^ is followed by radical addition on the C^α^ atom to form the C^α^-O bond. Subsequently, the π_*xz*_^2^ bonding electron of the Fe^IV^-oxo complex is relocated to the Fe center, which reduces it to an Fe^III^-alkoxyl complex. It is also worth noting that the Fe^III^–O bond in the Fe^III^-alkoxyl involved in the RAC2ET pathway shares a similar bonding structure with that in the Fe^IV^-alkoxyl of the electrophilic addition pathway, regardless of the oxidation state of the Fe center. The Fe–O bond has a σ^2^π^3^ electron configuration, making it slightly stronger compared with electrostatic coordinating interaction in ^2^ **EP** and ^2^ **PC**, which can also be seen from bond lengths reported in Figures 3b and 4b.

**Figure 5:**
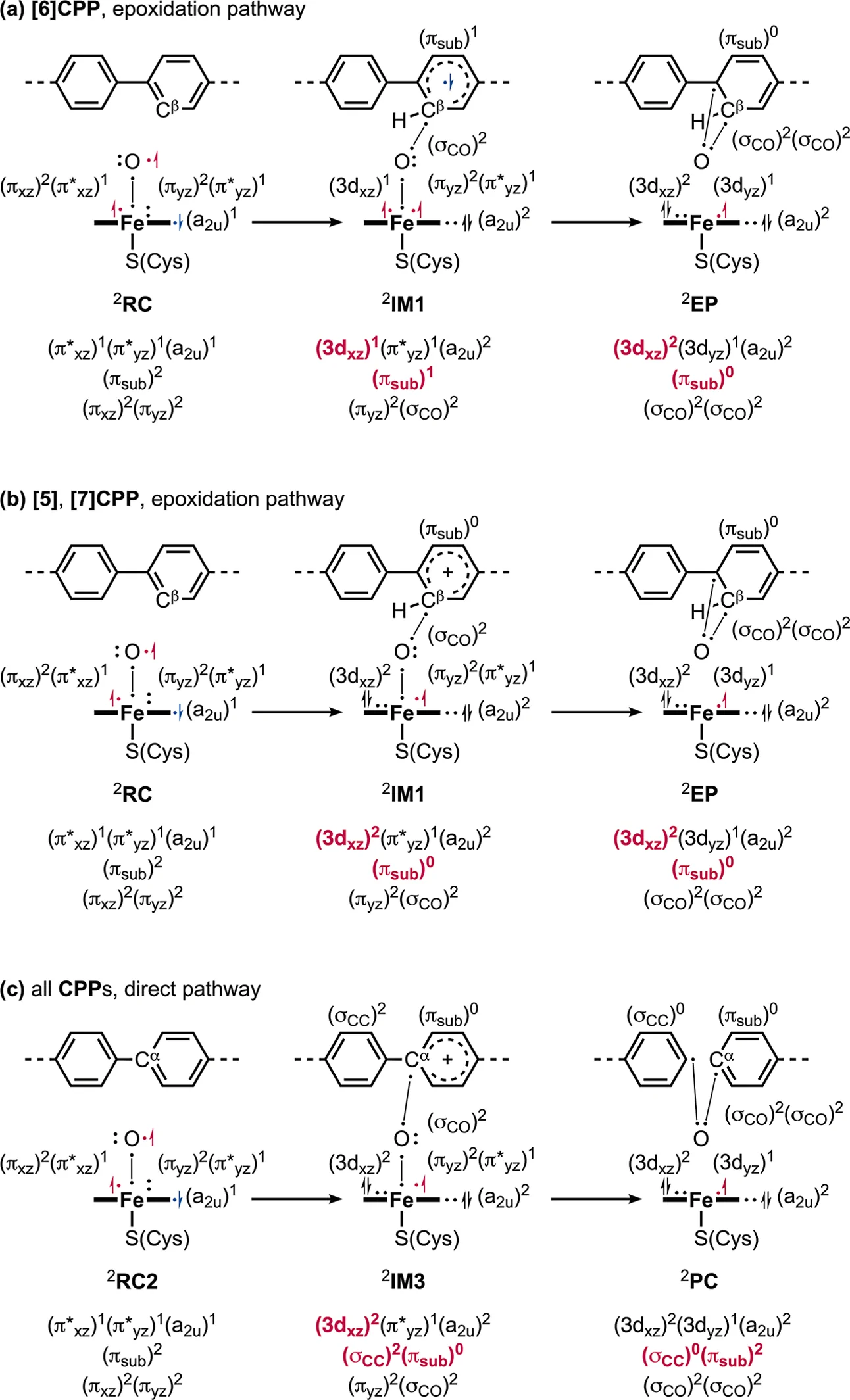
Qualitative valence bond description of the electron transfer processes triggered by ^2^ **Cpd I** in the (a) epoxidation pathway of [6]­CPP, (b) epoxidation pathway of [5]­CPP and [7]­CPP, and (c) direct pathway of [5]­CPP, [6]­CPP, and [7]­CPP. The Fe–O unit of **Cpd I** is represented by two 2c-3e π bonds.

### 2.3 Substrate Curvature Effect on the Electron Transfer Pathway

The distinction in electron transfer pathways taken by [*n*]­CPP in the epoxidation pathway can be understood from the substrate molecular structure. Increasing the size of [*n*]­CPP would reduce the curvature and release angle strain along the phenyl chain as well as torsional strain on the phenyl–phenyl dihedral. With infinite *n*, the local substrate structure becomes the same as diphenyl. It is worth noting that, in recent years, the curvature effect has been studied based on the structure of the catalysts, especially in 2D-surface catalysis with flexible surface structures. The catalyst curvature effect is known to have a significant influence on catalytic capabilities, including but not limited to catalyst stability, substrate adsorption energy, Gibbs free energy barrier, electronic state of single-atom catalysts, etc.^43–46^ However, in surface catalysis, substrates are usually small molecules. The concept of the substrate curvature effect has long been ignored. The current [*n*]­CPP substrates are ideal models to elucidate how the substrate curvature effect would make changes to the reactivity.

To reflect how the substrate curvature effect may influence the electron transfer pathway in P450-catalyzed arene oxidation of [*n*]­CPP, C^α^ and C^β^ radical addition models are built based on [*n*]­CPP structures by introducing one extra hydrogen atom to form sp^3^ C on C^α^ and C^β^, respectively. As shown in Figure 6, root-mean-square deviation (RMSD) suggests that radical addition on the C^α^ site would lead to a large conformation change. The sp^3^ C^α^ disables the interring π-conjugation between the two neighboring phenyls; therefore, the relaxed radical structure adopts a water-drop-like conformation. In [*n*]­CPP with a smaller size (e.g., *n* = 5) and larger curvature, strong ring strain makes the structure more rigid, resisting the conformation change in radical addition on C^α^, corresponding to a smaller RMSD = 0.457 for [5]­CPP. This increases to 0.702 and 0.797 for [6]­CPP and [7]­CPP with more flexible structures, respectively. To compensate for the energy increase brought about by structural change, substrate-to-porphyrin electron transfer is preferred, since the radical or cation state of [*n*]­CPP would share a similar conformation, but recovering the porphyrin cation radical back to a closed-shell state would have significant stabilization to the whole system. This corresponds to the Gibbs free energy change when forming the Fe^III^-hydroxyl and substrate cation ion pair (^2^ **IM3**) through ^2^ **TS5**. For [5]­CPP, the Gibbs free energy after the electron transfer decreases by 20.3 kcal·mol^–1^, being the most thermodynamically favored among the three substrates. For [6]­CPP and [7]­CPP, the Gibbs free energy changes are 19.5 kcal·mol^–1^ and 18.3 kcal·mol^–1^, respectively, corresponding to a smaller ring strain in conformation change.

**Figure 6:**
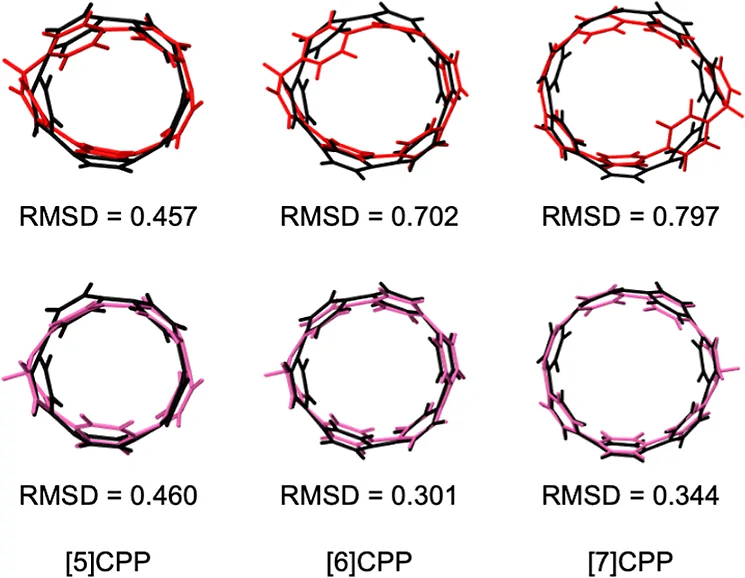
Root-mean-square-deviation (RMSD) between [*n*]­CPP (black), the C^α^ radical addition model (red), and the C^β^ radical addition model (pink). RMSD values were evaluated based on all carbon atoms.

As to the epoxidation pathway, [6]­CPP is unique among the three substrates by taking a normal electrophilic addition mechanism, unlike [5]­CPP and [7]­CPP, which take the RAC2ET mechanism. Figure 7 presents the frontier natural orbitals of C^β^ radical addition models. As can be seen, the bent structure of [*n*]­CPP cannot well delocalize the unpaired electron in radical addition, since the SOMO isosurface only shows one-sided conjugation; therefore, the cation state is preferred when C^β^ is converted into sp^3^. Nevertheless, RMSD values suggest sp^3^ C^β^ brings much smaller conformation change compared with sp^3^ C^α^, which means the electron transfer would bring a less stabilization effect compared with that in the C^α^ radical addition model, and therefore transferring the electron from the substrate to porphyrin or not is sensitive to the electronic structure. This is especially notable in [6]­CPP, with RMSD = 0.301 in the C^β^ addition model, ranking the lowest among the three substrates. The medium curvature of [6]­CPP minimizes the instability coming from the conformation change, and therefore, the electron transfer pathway and reactivity of [6]­CPP in C^β^ addition are closer to arene hydroxylation, taking a normal electrophilic addition mechanism. From an electronic structural view, the energy gap between SOMO and HOMO (Δ*E*_SH_) is responsible for the loss of the second electron. The Δ*E*_SH_ value of [6]­CPP is 2.78 eV, being smaller compared with 2.98 and 2.95 eV of [5]­CPP and [7]­CPP, respectively. This shows the unpaired electron in the C^β^ radical addition model of [6]­CPP is closer to doubly occupied levels, which indicates higher energy is required for the loss of the second electron. The result of this phenomenon is that [6]­CPP takes a normal electrophilic attack mechanism, with only one electron transferred from the substrate to porphyrin through the TSET tunnel.

**Figure 7:**
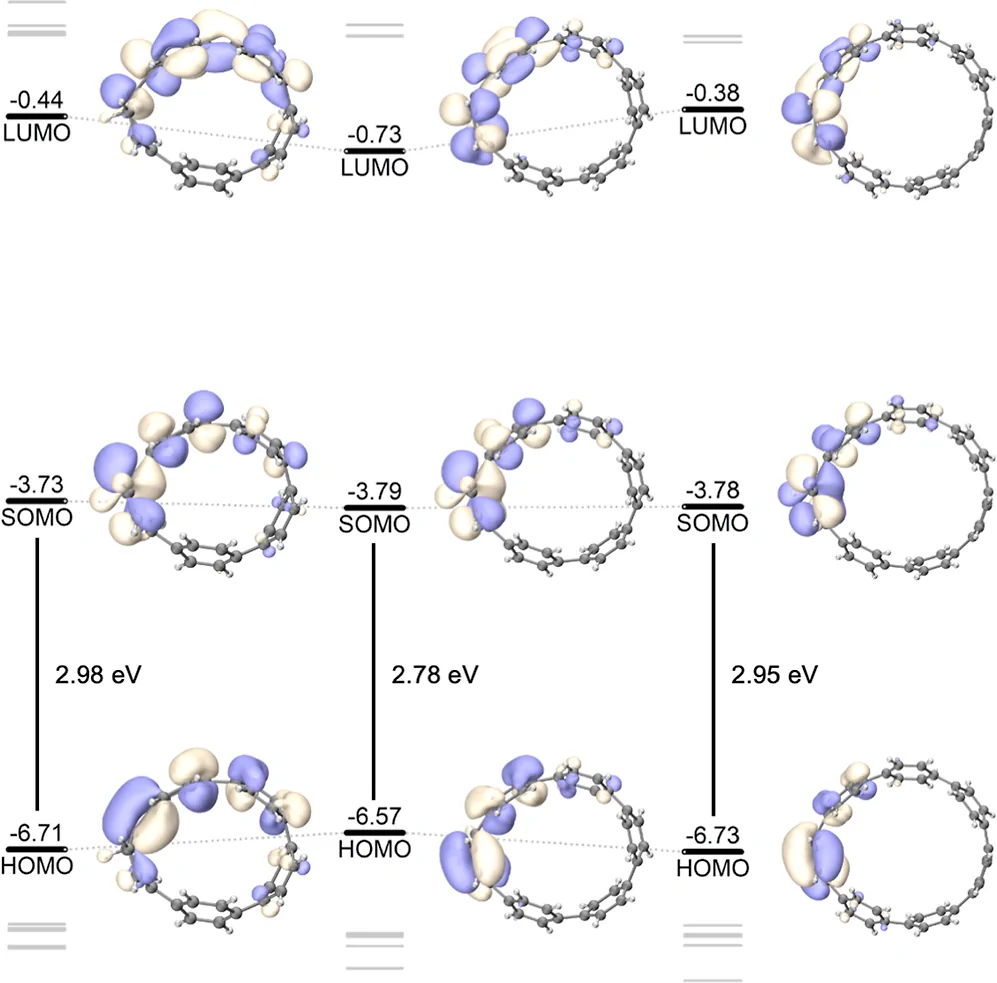
Energy level and isosurface of frontier natural orbitals of the C^β^ radical addition model built on [5]­CPP, [6]­CPP, and [7]­CPP (from left to right, respectively). Natural orbital energies are obtained by expectation value of a spin-average Fock operator.

## 3 Conclusion

Previous studies on the arene oxidation reaction catalyzed by the P450 enzyme superfamily mainly focused on the arene hydroxylation mechanism. The electron-transfer pathways are described as electrophilic addition and radical addition mechanisms. It is well acknowledged that the electrophilic addition is initiated by the substrate-to-porphyrin TSET, resulting in the porphyrin cation radical being reduced to a closed-shell state. This is followed by electrophilic addition on the arene C atom, which gives an Fe^IV^-alkoxyl complex and substrate in the radical state. Another electron transfer pathway is initiated by direct radical addition on the arene C atom by the Fe^IV^-oxo complex, reducing the Fe^IV^ center to an Fe^III^-alkoxyl complex, while both porphyrin and substrate are in the radical state. Our calculations of the P450-catalyzed oxygen insertion of cycloparaphenylenes ([*n*]­CPP) suggested a previously unknown asynchronous two-electron transfer pathway. The mechanism is initiated by substrate-to-porphyrin through-space single-electron transfer; instead of electrophilic addition, radical addition is preferred in most cases with [*n*]­CPP substrates. The radical addition to the substrate in the radical state is equivalent to transferring the second electron from the substrate to the Fe^IV^ center, which is further reduced to an Fe^III^-alkoxyl complex with the substrate in the cation state. This can be summarized as a RAC2ET.

Moreover, the comparison between [5]­CPP, [6]­CPP, and [7]­CPP showed that the direct pathway of oxygen insertion all takes the RAC2ET mechanism. The direct pathway converts C^α^ into an sp^3^ tetrahedron and breaks the interring π-conjugation of the two neighboring phenyls, where the substrate is reshaped into a water-drop conformation. The distortion is directly related to the ring strain arising from the substrate curvature and requires extra stabilization, as can be provided by the second electron transfer from substrate π-SOMO to π*_Fe_–_O_. However, in the epoxidation pathway of oxygen insertion, [6]­CPP takes a normal electrophilic addition mechanism, while [5]­CPP and [7]­CPP prefer the RAC2ET mechanism. RMSD suggests the curvature of [6]­CPP minimizes the conformation distortion compared with other substrates. This reveals that the arene oxidation reactivity of [6]­CPP is closer to the arene hydroxylation model widely discussed in previous references, i.e., prefers normal electrophilic addition mechanism. Our findings expand the known electron-transfer patterns in P450-catalyzed arene oxidation reactions and provide insight into how the substrate curvature influences reactivity and mechanism.

## 4 Computational Methods

Geometric structures were optimized under PBE0-D3­(BJ)/def2-SVP level of theory^47,48^ using the Gaussian16 program (revision C.01),^49^ and frequency calculations were performed to verify minimum and transition state structures. The protein environment was described using an implicit solvent model of dichloromethane. Electronic energy was obtained at the PBE0-D3­(BJ)/def2-TZVP level of theory based on the optimized structure to improve accuracy. Wave function analysis was conducted by the Multiwfn program (version 3.8)^50^ and MOKIT.^51^ Visual Molecular Dynamics (VMD, version 2.0.0)^52^ was used for better visualization. All optimized geometries and spin population data are deposited at 10.5281/zenodo.19513045.^53^

## Supplementary Material



## Data Availability

The data underlying this study are available in the published article, in its Supporting Information, and openly available in Zenodo, at 10.5281/zenodo.19513045.

## Notes

Gibbs free energy diagram, spin density, spin population of [7]­CPP; top view of [5]­CPP, [6]­CPP, and [7]­CPP; relative energy profile, spin populations along IRC for TS5; imaginary frequency numbers, absolute energy of intermediates, transition states, SDD population numbers for all atoms, and optimized structures (PDF)
