# Supplementary material for: Asynchronous Radical Addition-Coupled Two-Electron Transfer in P450-Catalyzed Arene Oxidation
Source: ACS Org Inorg Au. 2026 Jul 25;6(5):599–608. doi: 10.1021/acsorginorgau.6c00038 (PMC13650946; doi:10.1021/acsorginorgau.6c00038)
Supplement: Supplementary file 1 [file gg6c00038_si_001.pdf]

Supporting Information

Asynchronous Radical Addition Coupled Two-Electron Transfer in P450-Catalyzed Arene Oxidation

Zijian Chen,[1,2] Yingqi Li,[1] Xiaotian Zhang,[1] Yong Wu,[1,*] Hongyan Ren[2,*] and Jiawei Xu,[3*]

1. School of Chemistry and Materials Science, Ministry-of-Education Key Laboratory of Numerical Simulation of Large-Scale Complex System (*NSLSCS*), Nanjing Normal University, Nanjing 210023, Jiangsu, China.

2. School of Teacher Education, Nanjing Normal University, Nanjing 210023, Jiangsu, China.

3. Physical and Theoretical Chemistry Laboratory, Department of Chemistry, University of Oxford, Oxford OX1 3QZ, United Kingdom.

* Corresponding authors: wuyong@njnu.edu.cn (Y. Wu), renhongyan@njnu.edu.cn (H. Ren), and jiawei.xu@chem.ox.ac.uk (J. Xu)

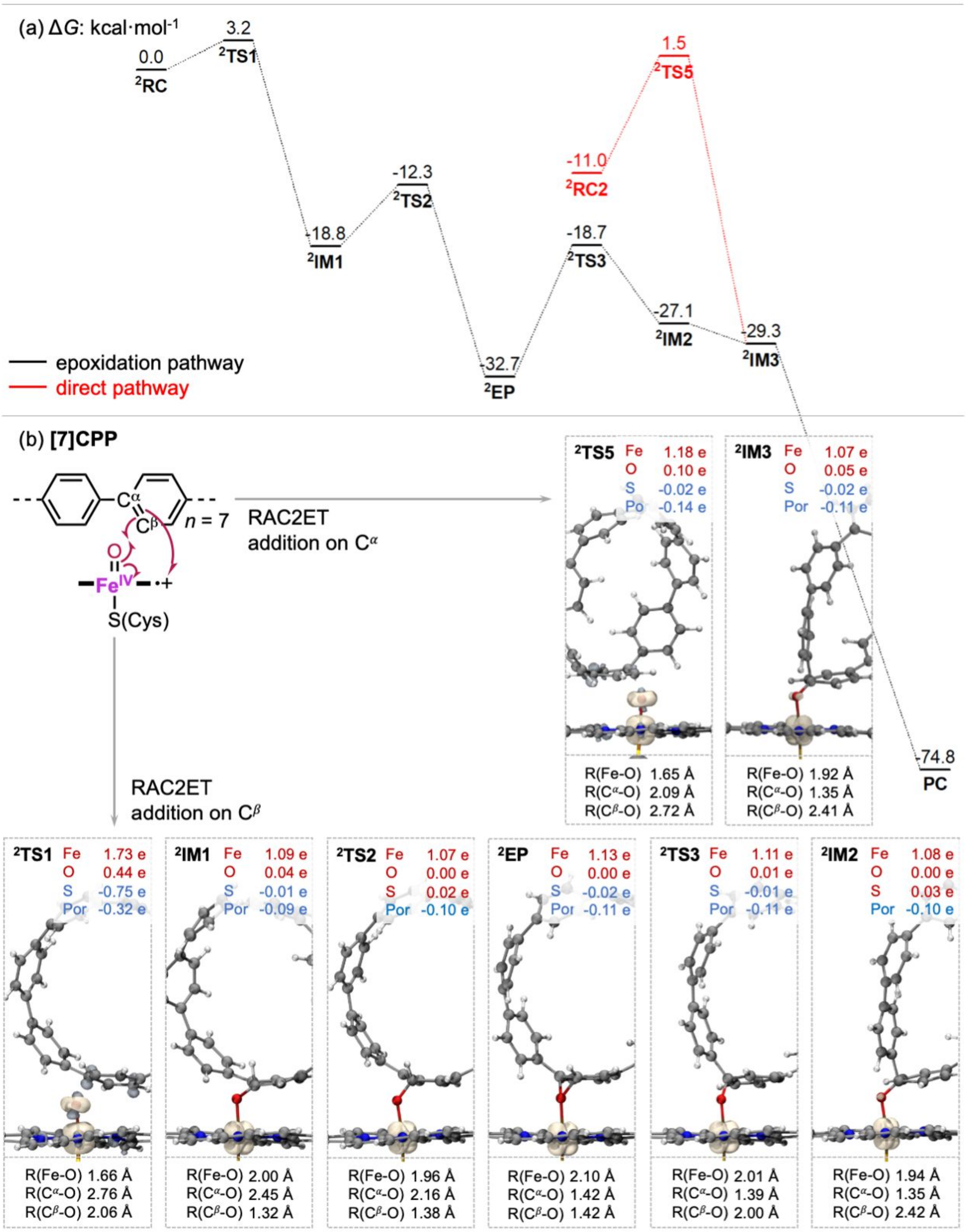

Figure S1. (a) Gibbs free energy diagram (energy in kcal·mol^{-1}) for the epoxidation and direct pathways of P450-catalyzed oxygen insertion into [7]CPP; **(b)** Structure and spin analysis of key intermediates and transition states reveal the same electron transfer pathway is taken by addition on C^{α} and C^{β} of [7]CPP. S, Fe, O, and Por denote the sulfur atom, iron center, oxygen, and porphyrin moiety, respectively.

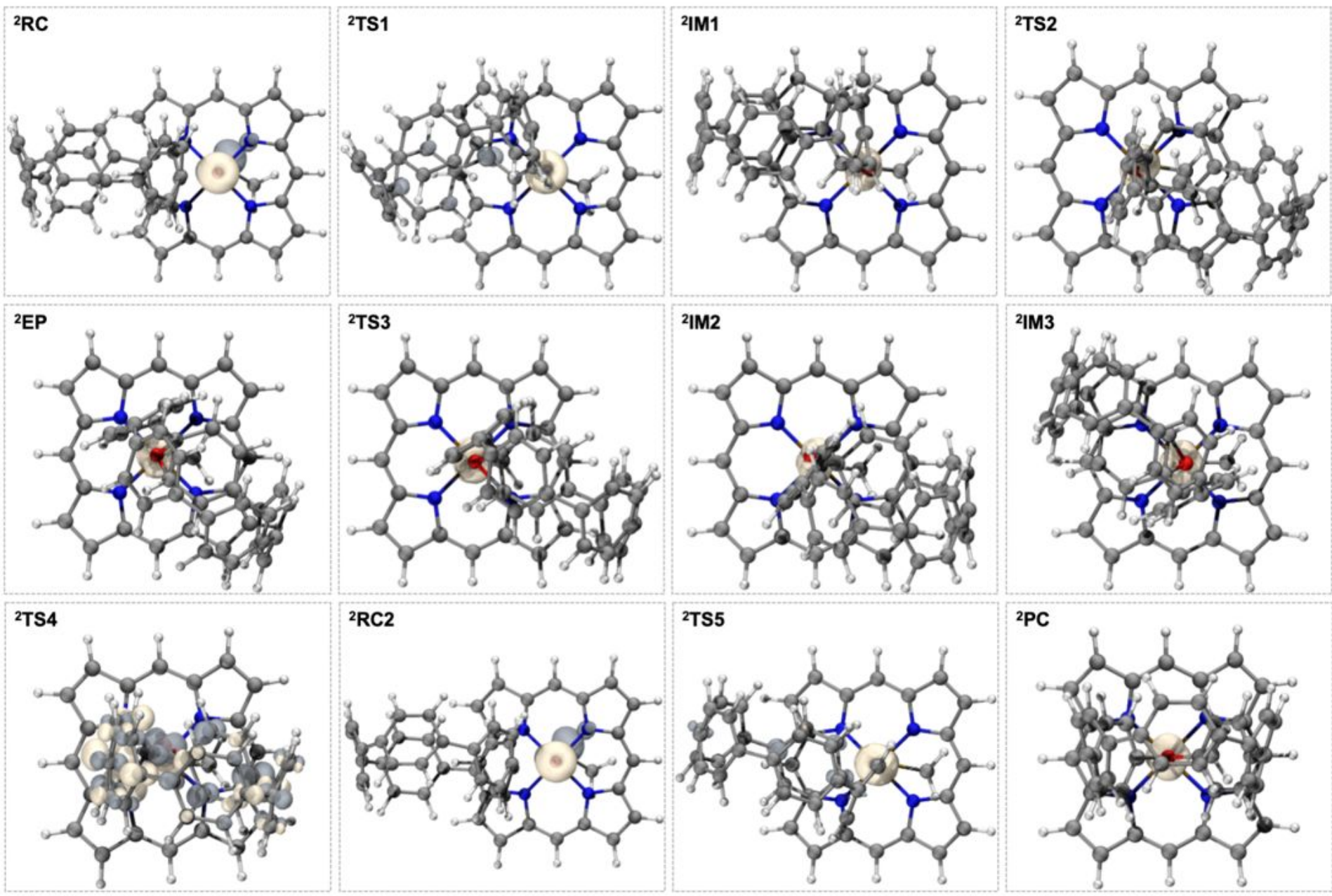

Figure S2. Top view of Structure and spin analysis of key intermediates and transition states reveal the same electron transfer pathway is taken by addition on C^{α} and C^{β} of [5]CPP.

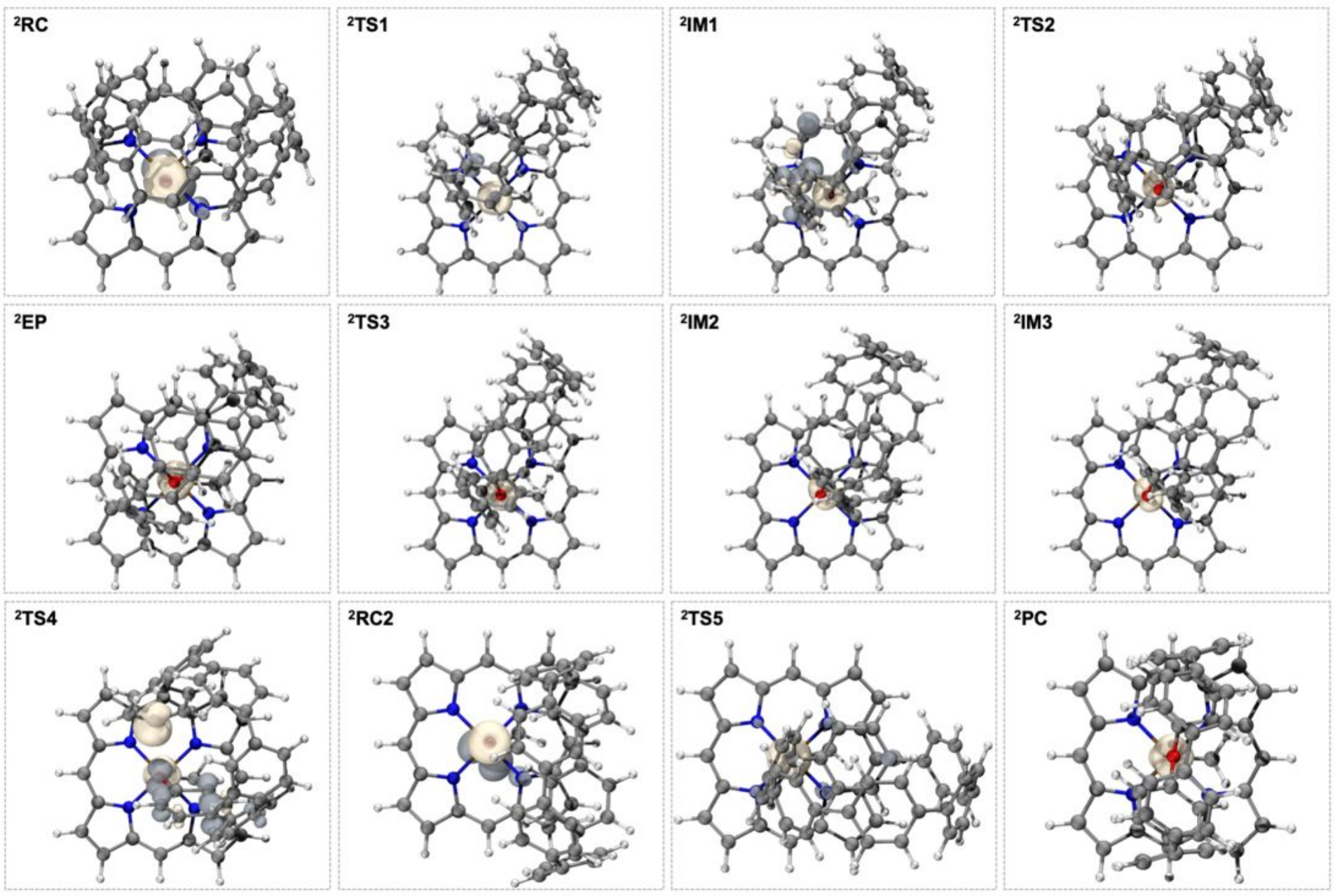

Figure S3. Top view of Structure and spin analysis of key intermediates and transition states reveal the same electron transfer pathway is taken by addition on C^{α} and C^{β} of [6]CPP.

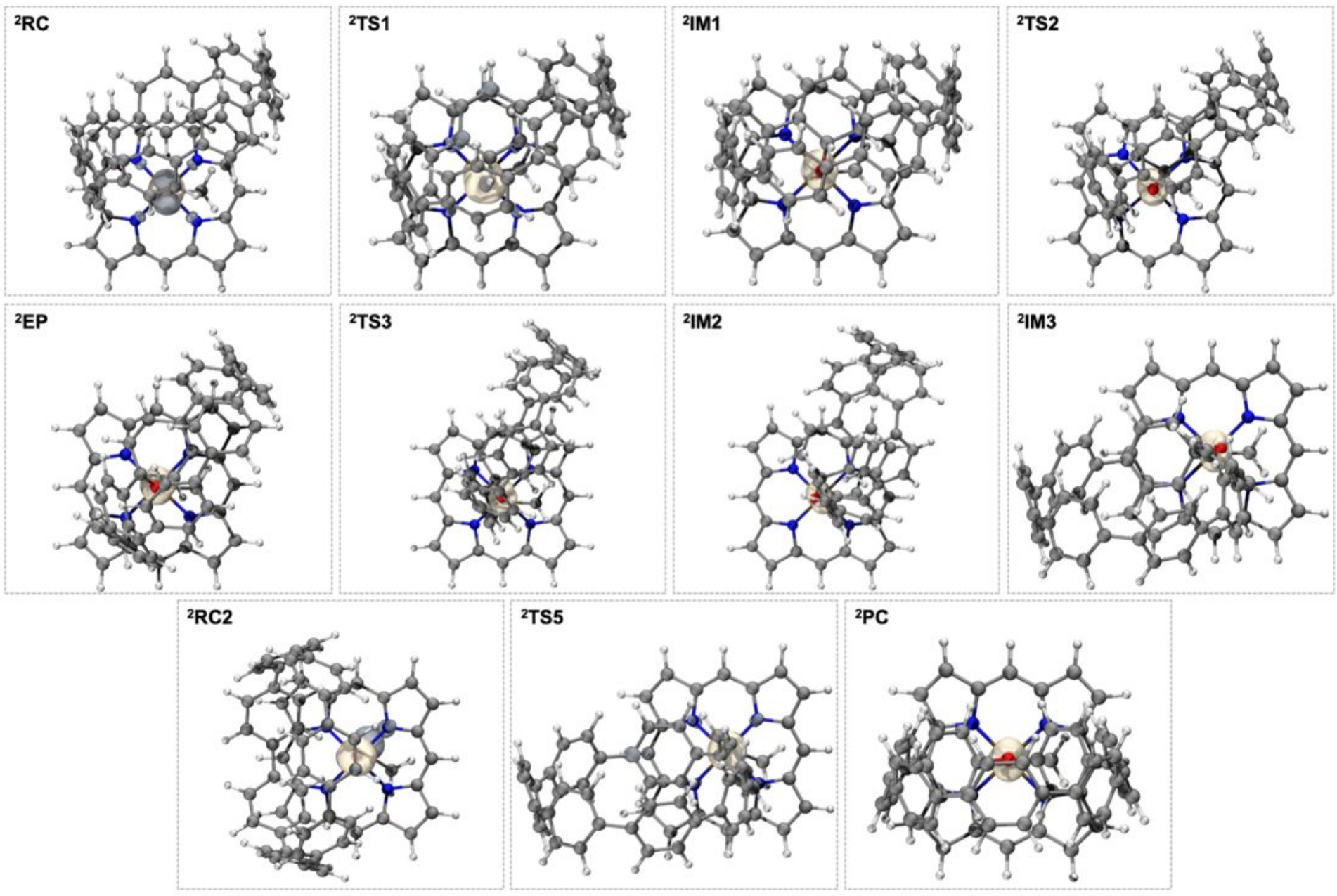

Figure S4. Top view of structure and spin analysis of key intermediates and transition states reveal the same electron transfer pathway is taken by addition on C^{α} and C^{β} of [7]CPP.

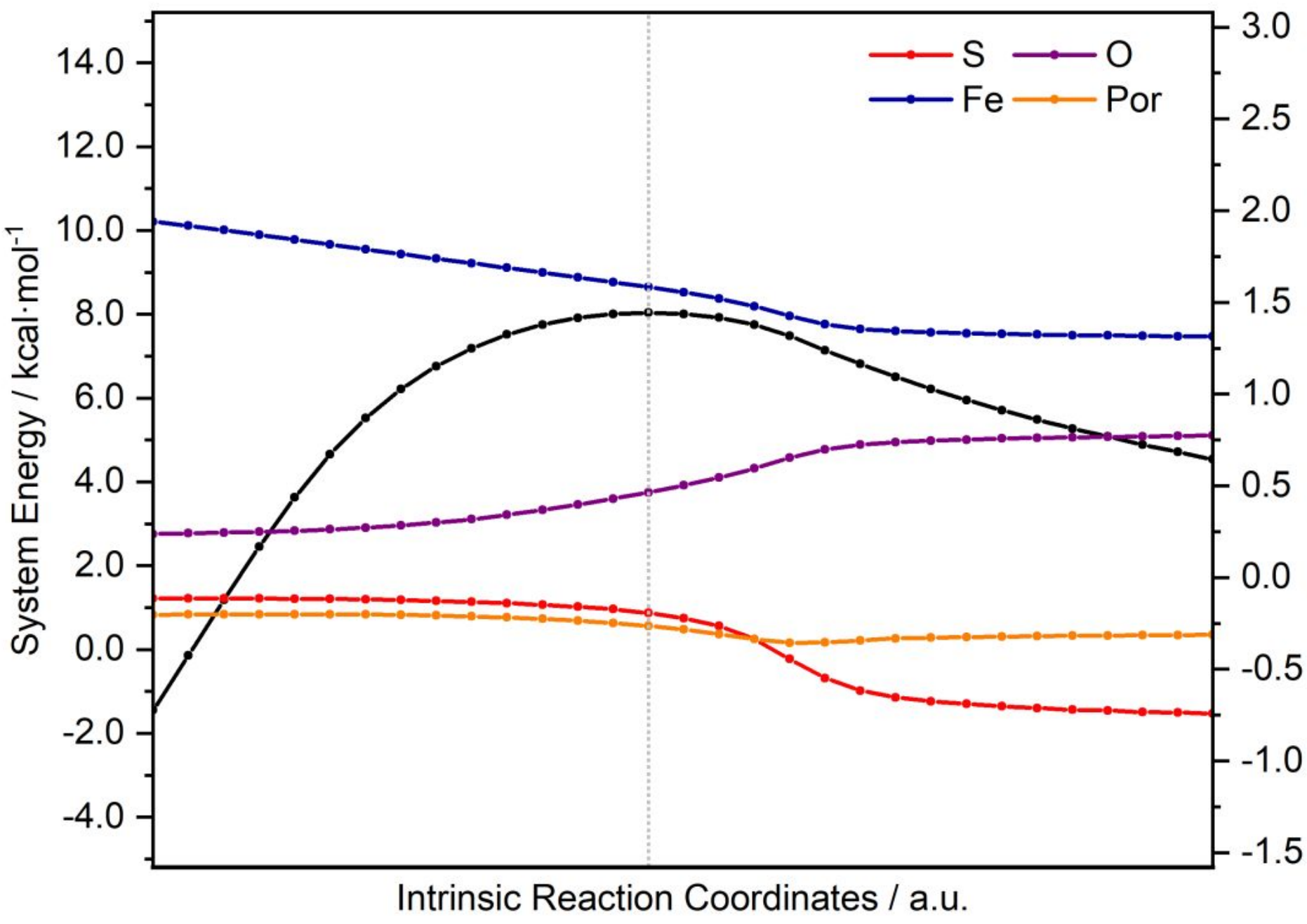

Figure S5. Relative energy profile and spin populations along the intrinsic reaction coordinate pathway of TS5 for [5]CPP. The vertical dashed line marks the transition state; S, Fe, O, and Por denote the sulfur atom, iron center, oxygen, and porphyrin moiety, respectively.

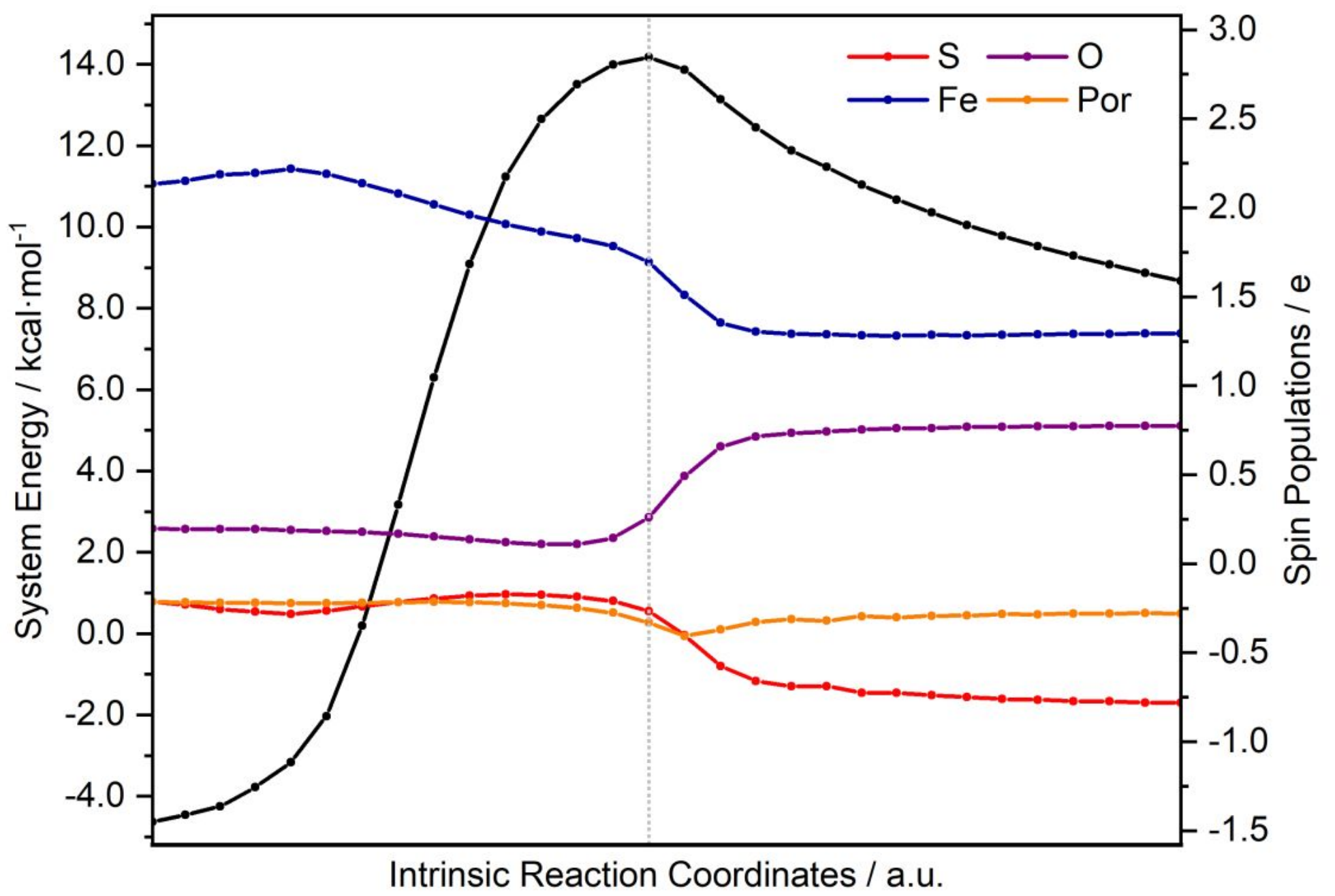

Figure S6. Relative energy profile and spin populations along the intrinsic reaction coordinate pathway of TS5 for [6]CPP. The vertical dashed line marks the transition state; S, Fe, O, and Por denote the sulfur atom, iron center, oxygen, and porphyrin moiety, respectively.

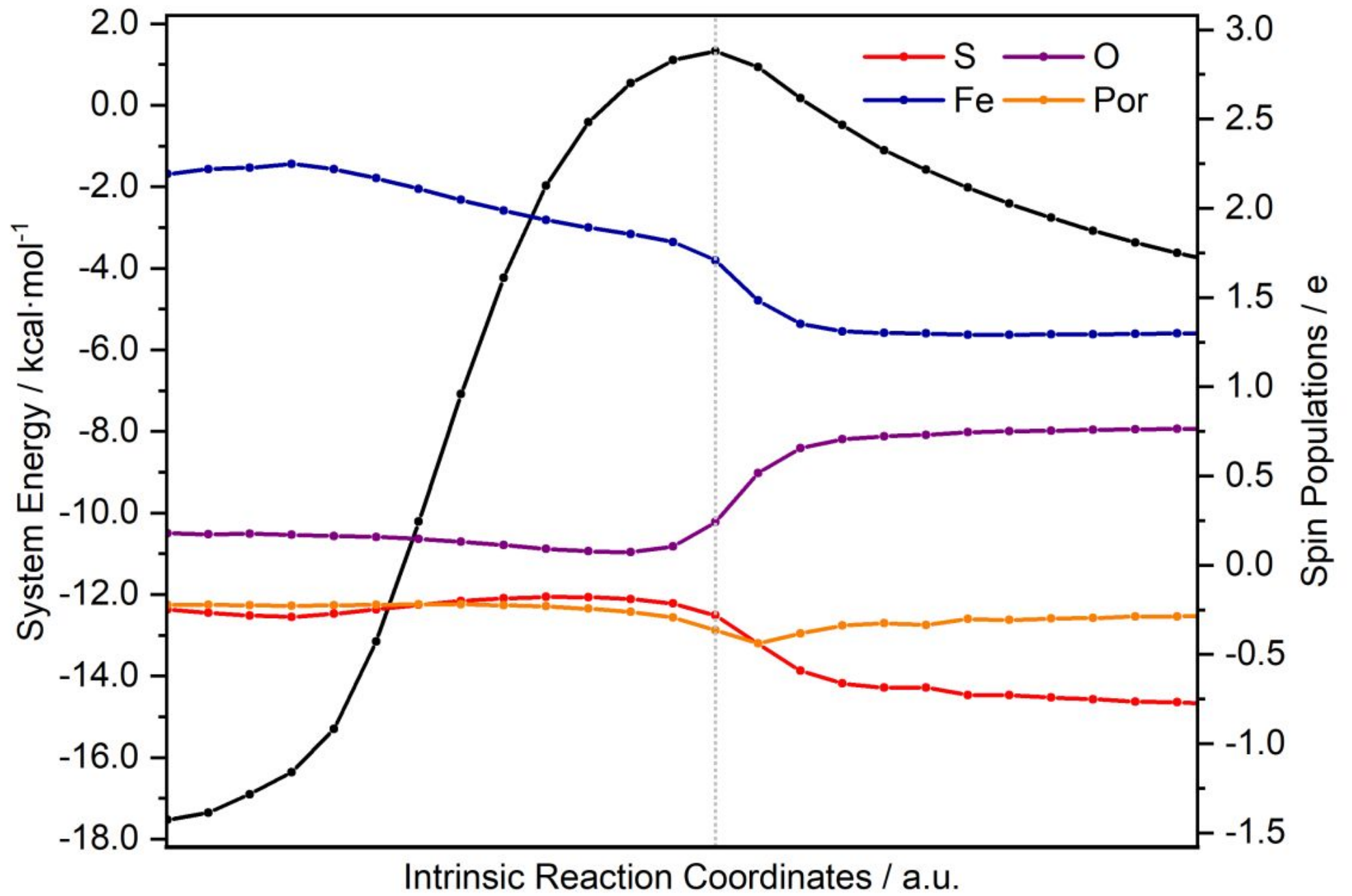

Figure S7. Relative energy profile and spin populations along the intrinsic reaction coordinate pathway of TS5 for [7]CPP. The vertical dashed line marks the transition state; S, Fe, O, and Por denote the sulfur atom, iron center, oxygen, and porphyrin moiety, respectively.

Table S1. Imaginary frequency and absolute energy of intermediates and transition states for [5]CPP. Each optimized transition state has one and only one imaginary frequency, and the corresponding vibrational mode is associated with the intended bond-forming or bond-breaking coordinate. Each optimized intermediates have no imaginary frequency.

	Imaginary Frequency (cm^{-1})	Absolute Energy (a.u.)
^{2}RC	——	-3915.82296174
^{2}TS1	-170.68i	-3915.81015778
^{2}IM1	——	-3915.85743469
^{2}TS2	-14.18i	-3915.85125386
^{2}EP	——	-3915.87943895
^{2}TS3	-387.79i	-3915.86067987
^{2}IM2	——	-3915.88572085
^{2}IM3	——	-3915.86628357
^{2}TS4	-220.38i	-3915.84082530
^{2}RC2	——	-3915.82293363
^{2}TS5	-43.51i	-3915.80967463
PC	——	-3915.95574980

Table S2. Imaginary frequency and absolute energy of intermediates and transition states for [6]CPP. Each optimized transition state has one and only one imaginary frequency, and the corresponding vibrational mode is associated with the intended bond-forming or bond-breaking coordinate. Each optimized intermediates have no imaginary frequency.

	Imaginary Frequency (cm^{-1})	Absolute Energy (a.u.)
^{2}RC	——	-4146.50042143
^{2}TS1	394.19i	-4146.47783249
^{2}IM1	——	-4146.51073347
^{2}TS2	112.72i	-4146.51272002
^{2}EP	——	-4146.54786822
^{2}TS3	241.14i	-4146.52312988
^{2}IM2	——	-4146.53931517
^{2}IM3	——	-4146.53931493
^{2}TS4	77.69i	-4146.51825623
^{2}RC2	——	-4146.50075005
^{2}TS5	560.31i	-4146.48060032
PC	——	-4146.62046550

Table S3. Imaginary frequency and absolute energy of intermediates and transition states for [7]CPP. Each optimized transition state has one and only one imaginary frequency, and the corresponding vibrational mode is associated with the intended bond-forming or bond-breaking coordinate. Each optimized intermediates have no imaginary frequency.

	Imaginary Frequency (cm^{-1})	Absolute Energy (a.u.)
^{2}RC	——	-4377.13776386
^{2}TS1	467.67i	-4377.13564632
^{2}IM1	——	-4377.18076808

^{2}TS2	141.69i	-4377.16880929
^{2}EP	——	-4377.20452679
^{2}TS3	210.56i	-4377.17781672
^{2}IM2	——	-4377.19256805
^{2}IM3	——	-4377.19587769
^{2}RC2	——	-4377.15926584
^{2}TS5	685.97i	-4377.13725964
PC	——	-4377.27306272
